# Early Marriages: Royal and Otherwise

**Published:** 1895-10-12

**Authors:** 


### Early Marriages: Royal and Otherwise.

According to the annual report of the Registrar-General for 1893, there were married in England during that year 39,457 girls who were under age. Men, however, do not rush into matrimony quite so early, the number of men married under age being but 12,257. Taken together, however, the number is large, and is suggestive of a mass of misery and wretchedness which is immeasurable and can be imagined better than described.

Clouston says: "The young man who marries before his beard is fairly grown breaks a law of nature and sins against posterity," while the late Dr. Matthews Duncan says: "Below 20 years of age woman is immature; she runs considerable risk of proving sterile; and if she does bear a child, she runs a comparatively high risk of dying in childbed; besides, her early marriage brings other disadvantages."

On this subject, probably, M. Joseph Korosi, of the Buda Pesth Statistical Bureau, has made more extensive inquiries than any recent investigator, and his conclusions are to the effect that immature parents bring forth a degenerate stock in which the percentage of idiots, cripples, consumptives, criminals, &c., is immensely larger than in the children of those who are mature. In a clinical lecture delivered in 1889, Dr. Matthews Duncan laid it down that in woman the age of maturity is from 20 to 25, and he went on to say, "At this age woman has the lowest risk of sterility, the greatest likelihood of having healthy children that will long survive, the greatest likelihood of herself surviving childbirth, the lowest risk of having abortions, of having excessive family, of having plural pregnancy, and of bringing forth idiots."

That misery results from early marriages everyone acknowledges; but it is common to attribute them to the fact that so many of those who marry recklessly are poor, and to say that the evil consequences of early marriage are due to the want and privation, the improvidence and the ignorance, which prevail among the majority of those who make this great mistake in life, rather than to anything wrong in the act itself. These quotations, however, lift the question out of the realm of economics, for besides condemning themselves and their children to poverty and all its evils, it is clear that those who marry before they are mature condemn their offspring to degeneracy.

It must not be forgotten also that the married state is one of considerable danger to the immature. Farr says, "Under the French law young men of the age of 18, and young women of the age of 15, can legally marry. Of the few young married pairs living the mortality in both husbands and wives was excessively high under the age of 20. Twice as many wives under 20 died in the year as died out of the same number of the unmarried; and the mortality was much higher than it was among husbands and wives in the subsequent decennial of life." ("Vital Statistics," page 439.) But as regards girl mothers there are special dangers. We need not now enter into details in regard to this, a fact which is well recognised, but may content ourselves by stating, on the authority of Dr. Farr, that while in the aggregate, derived from a large number of observations, nearly 1 in every 189 accouchements at all ages were fatal, this proportion rose among those between 15 and 25 to 1 in 149, while it sank between 25 and 35, the great child-bearing age, to 1 in 235.

Reverting, however, to the effect of early marriages on the offspring, it is to be noted that what we have stated not only receives support from the writings of Shakespeare and Aristotle, but accords with the experience of breeders of cattle and other domestic animals. For example, it is a common practice in many districts to destroy the first litter of puppies, because they are looked upon as sickly, lacking in pluck, and never coming to anything; while as regards other animals which are more under control, such as sheep and cattle, care is always taken never to use them for breeding purposes until they are mature, for not only is early breeding found unremunerative from the greater mortality among the offspring, but it is found that the cow which has a calf too young never comes to the size and strength which she otherwise would have done.

The law of England permits the marriage of boys of 14 with girls of 12, but the common sense of the community has brought it about that the average age of first marriages is 26·55 for men and 25·04 for women; or, taking five year periods, that the largest number of men marry between 25 and 30, while the largest number of women marry between 20 and 25.

The average age at which women in the different classes of society marry is not without interest, varying as it does, according to Newsholme, from 22·46 among miners to 26·4 among the professional and independent classes. It would appear that early marriages are to be looked upon as resulting largely from want of self-control, and it is satisfactory to note that since the year 1874, when they reached their highest point, these child marriages have been steadily decreasing in frequency. It is then with some anxiety and surprise that we note that some branches of the Royal Family seem to separate themselves from the educated classes and to join the proletariate in the perpetuation of this evil custom of early marrying.

It is not necessary to go far for examples. H.R.H. the Empress Frederick was married when under 18 years of age, while one of her daughters was married when aged seventeen years and seven months. Of the daughters of the Duke of Edinburgh, one was married when seventeen years and three months, and another when seventeen years and five months old; and now we hear of the engagement of the Princess Alexandra, who was seventeen years old on Sept. 1. It is difficult to look without apprehension at such early marriages. It may often be desirable for State reasons that an amount of intermarrying should take place in royal families which would be regarded with disapproval in less exalted positions; but all the more important is it that an age for marriage should be chosen which should not only give greater opportunities for personal selection, but should accord with what we know is the best for the perpetuation of a sound and healthy race.

